# 

**Published:** 1914-05

**Authors:** 


### CONTENTS.

ORIGINAL COMMUNICATIONS—

Six Roetgenograms Illustrating some Commoner Gastro-Intestinal Disorders, by George M. Niles, M. D., Atlanta, Ga. .......... 49

The Essentials in the Equipment of a Hospital Laboratory, by Allen H. Bunce, A. B., M. D. .......... 56

The Present States of Pituitrin in Obstetrics, By James Robert McCord, M. D., Atlanta, Ga. .......... 60

Treatment of Rheumatism with Phylacogen (Report of cases), by W. C. Knnedy, M. D., Talmo, Ga. .......... 64

Fulton County Medical Society, Regular Meeting May 7, 1914. By R. R. Daly, M. D. .......... 71

EDITORIAL .......... 76

Selctions and Abstracts .......... 78

